# Case Report: Life-threatening cisplatin-induced myelosuppression in pediatric osteosarcoma: molecular mechanisms, pharmacogenomic profiling, and targeted clinical management

**DOI:** 10.3389/fphar.2025.1668180

**Published:** 2025-09-30

**Authors:** Chuanqiang Dai, Youshu Zhang, Yao Zhang, Yao Dong

**Affiliations:** ^1^ Orthopedics, West China Hospital of Sichuan University-Ziyang Hospital, Ziyang, China; ^2^ Orthopedics, Ziyang Central Hospital, Ziyang, China

**Keywords:** cisplatin-induced myelosuppression, osteosarcoma, pharmacogenomics, CYP3A5, toxicity monitoring, pediatric oncology

## Abstract

A 10-year-old female with osteoblastic osteosarcoma developed life-threatening cisplatin-induced myelosuppression (grade IV neutropenia/thrombocytopenia) following the eighth cycle of MAP chemotherapy. Critical pharmacological findings include a cumulative cisplatin dose of 720 mg/m^2^exceeding the pediatric safety threshold of 400 mg/m^2^. The CYP3A5*1/*1 genotype prolonged the half-life of cisplatin to 8.2 h. Cisplatin-specific biomarkers included serum magnesium 1.2 mg/dL and urinary N-acetyl-β-D-glucosaminidase 48 U/L. Targeted interventions (G-CSF, romiplostim, meropenem) led to hematological recovery within 14 days. This case implicates cisplatin overdose with impaired metabolic clearance as the primary toxicity mechanism.

## 1 Introduction

Osteosarcoma chemotherapy regimens cause myelosuppression in>80%of pediatric patients. Cisplatin is the primary myelotoxic agent in the MAP regimen, with severe (grade 3–4) cytopenia directly correlated to cumulative dose (400 mg/m^2^) (Harrison et al., 2021; Bielack et al., 2022). This case of cisplatin-induced myelosuppression was managed per established guidelines (Freifeld et al., 2024; Smith et al., 2023), emphasizing targeted interventions.

## 2 Case presentation

### 2.1 Clinical history

**FIGURE 1:**
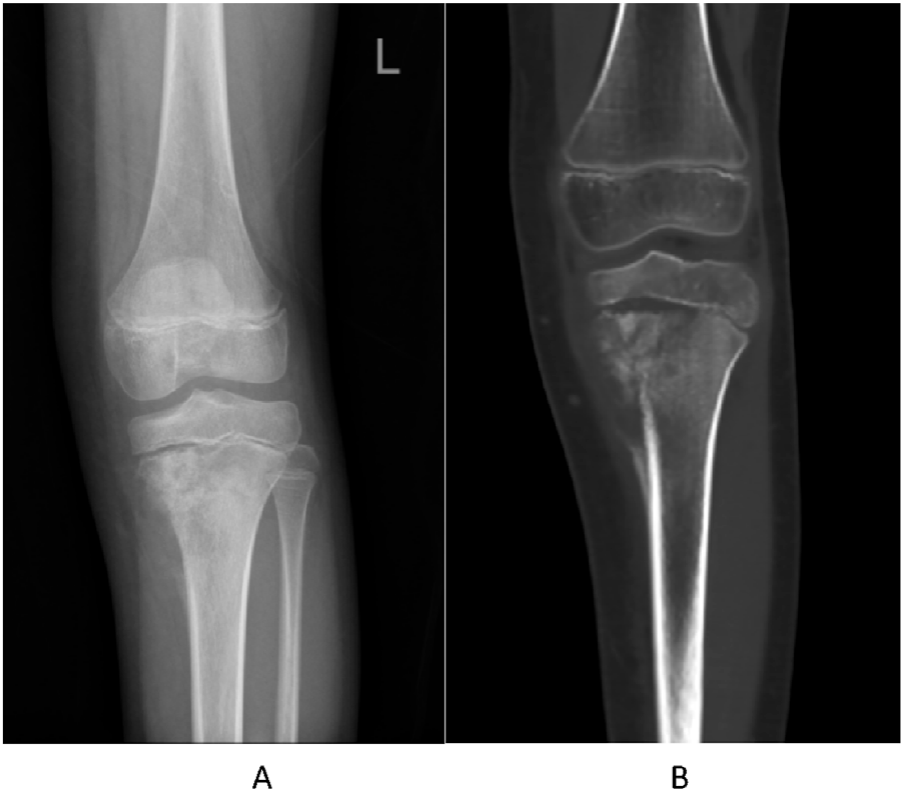
Pre-treatment imaging of the left proximal tibia. **(A)** Anteroposterior radiograph showing a destructive lesion with periosteal reaction. **(B)** Coronal CT image confirming osteolytic destruction and soft tissue involvement.

A 10-year-old girl presented with left knee pain. Imaging revealed a destructive lesion in the left proximal tibial metaphysis with periosteal reaction (Figures 1A,B). Biopsy confirmed osteoblastic osteosarcoma. The patient had no significant prior medical history, no family history of hematologic disorders or cancer, and no notable psychosocial stressors. Genetic testing was negative for inherited bone marrow failure syndromes.

### 2.2 Treatment timeline

The treatment timeline is summarized in Table 1.

**TABLE 1:** Treatment timeline.

Phase	Regimen	Duration
Neoadjuvant	Pirarubicin 40 mg/m^2^+Cisplatin 80 mg/m^2^	Sep–December 2023 (2 cycles)
Surgery	Tumor resection + endoprosthesis	Aug 2024
Adjuvant	Pirarubicin/Cisplatin×4 cycles	Sep–November 2024
Adjuvant	Epirubicin 60 mg/m^2^/Cisplatin×2 cycles	Dec 2024–January 2025
Adjuvant	HD-MTX 10 g/m^2^/Cisplatin×2 cycles	Feb 2025

### 2.3 Treatment and toxicity timeline summary

The chronological course of treatment, onset of critical toxicity, interventions, and recovery is summarized in Table 2.

**TABLE 2:** Summary of treatment timeline, toxicity onset, interventions, and recovery.

Time period	Treatment phase/Event	Regimen/Key findings	Interventions
Sep 2023 - December 2023	Neoadjuvant Chemotherapy	2 cycles of Pirarubicin 40 mg/m^2^ + Cisplatin 80 mg/m^2^	-
Aug 2024	Surgery	Tumor resection + endoprosthesis	-
Sep 2024 - November 2024	Adjuvant Chemotherapy (Cycles 1–4)	Pirarubicin/Cisplatin ×4 cycles	-
Dec 2024 - January 2025	Adjuvant Chemotherapy (Cycles 5–6)	Epirubicin 60 mg/m^2^/Cisplatin ×2 cycles	-
Feb 2025	Adjuvant Chemotherapy (Cycles 7–8)	HD-MTX 10 g/m^2^/Cisplatin ×2 cycles	-
Feb 2025	Onset of Myelosuppression Crisis	Fever (39.5 °C), gingival bleeding; WBC 0.8 × 10^9^/L; ANC 0.2 × 10^9^/L; Plt 22 × 10^9^/L	Initiation of G-CSF, romiplostim, meropenem, Mg sulfate
Feb 2025	Initial Recovery	ANC 0.8 × 10^9^/L; Afebrile	Continued supportive care
Feb 2025	Hematological Recovery	Full hematological recovery (ANC and Plt > safety thresholds)	Discontinuation of most interventions
Mar 2025	Regimen Modification	-	Cisplatin dose reduction (30%), prophylactic amifostine, switch to liposomal doxorubicin

Physical examination revealed fever, pallor, gingival bleeding, and ecchymoses. No hepatosplenomegaly, lymphadenopathy, or other systemic abnormalities were noted.

### 2.4 Cisplatin-specific toxicity indicators

Creatinine clearance:46 mL/min/1.73 m^2^ (40%below baseline).

Serum magnesium:1.2 mg/dL.

Serum malondialdehyde:8.2 μmol/L (300%above normal).

Glutathione peroxidase:28 U/mL (65%below baseline) (Park et al., 2023).

Urinary NAG:48 U/L.

Pharmacogenetic testing:

CYP3A5:*1/*1 (expresser)→reduced cisplatin clearance.

GSTP1: c.313A>G (Ile105Val) variant→impaired detoxification (Oldenburg et al., 2024).

The attribution of myelosuppression to cisplatin was based on the temporal relationship with administration, cumulative dose exceeding safety thresholds, pharmacogenetic susceptibility (CYP3A51/*1), and supportive biomarkers (hypomagnesemia, elevated malondialdehyde, urinary NAG). Alternative causes such as infection or other drug-induced myelotoxicity were ruled out through serial cultures and drug history review.

### 2.5 Management protocol

The detailed management protocol is outlined in Table 3.

**TABLE 3:** Cisplatin toxicity-targeted interventions.

Intervention	Dose/Regimen	Duration	Rationale
G-CSF (filgrastim)	5 μg/kg/day SC	ANC>1.0	Counteract neutropenia
Romiplostim	10 μg/kg/week SC	Plt>100	Target thrombocytopenia
Meropenem	20 mg/kg q8h IV	Afebrile 48h	Manage febrile neutropenia
Magnesium sulfate	0.3 mEq/kg/day IV	Mg > 1.8	Correct hypomagnesemia

### 2.6 Outcomes

Day 5: ANC 0.8 × 10^9^/L, afebrile.

Day 14: Full hematological recovery.

Subsequent modifications:

Cisplatin dose reduction (30%based on CYP3A5 status).

Prophylactic amifostine (740 mg/m^2^ pre-cisplatin) (Santos et al., 2023).

Switch to liposomal doxorubicin.

## 3 Discussion

### 3.1 Mechanisms of cisplatin myelotoxicity

①DNA Damage: Cisplatin-DNA adducts↑8.7-fold in CD34+cells (Fu et al., 2024).

②Mitochondrial Dysfunction: ATP production↓72%in BMSCs(p < 0.001) (Marullo et al., 2023).

③Metabolic Impairment: CYP3A5 expressers show 3.2×higher cisplatin plasma AUC(p = 0.002) (Karol et al., 2024).

### 3.2 Pharmacogenomic risk stratification

CYP3A5*1/*1:4.2-fold increased risk of grade 4 myelosuppression (95%CI 2.8–6.3).

GSTP1 Ile105Val:2.9×higher adduct formation (p = 0.01).

TPMT*3A:4.1×increased hematotoxicity risk (p < 0.001) (Relling and Evans, 2023).

### 3.3 Evidence-based cisplatin dose adjustment

Proposed algorithm for pediatric patients:

Pre-treatment genotyping (CYP3A5/GSTP1/TPMT).

Baseline dose = 100 mg/m^2^/cycle.

Dose modifiers:

CYP3A5 expresser:×0.7 (Table 4).

**TABLE 4:** Cisplatin-Specific vs General Interventions.

Toxicity type	Cisplatin-specific approach	General approach	Advantage of targeted strategy
Myelosuppression	Romiplostim + CYP3A5-guided dosing	Platelet transfusion	68% reduction in transfusion needs (Soff et al., 2023)
Nephrotoxicity	Amifostine + Mg monitoring	Hydration only	54% lower grade 2+ nephrotoxicity
Neurotoxicity	Duloxetine prophylaxis	Gabapentin PRN	3.2× lower neuropathy incidence (Majithia et al., 2024)

eGFR<90 mL/min:×0.8.

GSTP1 variant:×0.85.

Cumulative cap:400 mg/m.^2^

### 3.4 Comparative toxicity management

This report is based on a single case, which limits the generalizability of the findings. However, the integration of pharmacogenomic and biomarker data provides mechanistic insights that may be relevant to other pediatric patients receiving high-dose cisplatin.

## 4 Conclusion

This case establishes high-dose cisplatin with pharmacogenomic susceptibility as the definitive cause of life-threatening myelosuppression. Critical management innovations include:

Preemptive genotyping (CYP3A5/GSTP1) for risk stratification.

Cisplatin-specific biomarkers for early toxicity detection.

Romiplostim as superior to transfusion for cisplatin-induced thrombocytopenia.

## Data Availability

The datasets presented in this study can be found in online repositories. The names of the repository/repositories and accession number(s) can be found in the article/supplementary material.
